# Reported survival with severe mixed acidosis and hyperlactemia after toluene poisoning

**DOI:** 10.4103/1658-354X.76474

**Published:** 2011

**Authors:** Amr S. Omar, Masood ur Rahman, Said Abuhasna

**Affiliations:** *Department of Critical Care Medicine, Tawam Hospital/ John Hopkins Medicine, Al Ain, UAE*

**Keywords:** *Lactic acidosis*, *toluene*, *hyperlactemia*

## Abstract

Lactic acidosis is a recognized complication of the inhalant abuse such as toluene, especially in patients with renal insufficiency. We report a case of severe metabolic acidosis and hyperlactemia due to toluene sniffing. The favorable outcome, despite extremely poor clinical symptoms, signs, laboratory and radiological findings, was unexpected. Specific aspects of the clinical course are addressed. Toluene sniffing should be considered in evaluating sever metabolic acidosis. Favorable outcome could be achieved with early diagnosis and proper interventions.

## INTRODUCTION

Toluene (methylbenzene, toluol, phenylmethane) is an aromatic hydrocarbon (C_7_H_8_) commonly used as an industrial solvent for the manufacturing of paints, chemicals, pharmaceuticals and rubber. Solvent abuse is prevalent around the world since solvents are easily accessible and inexpensive.[1] We report a case of severe mixed acidosis and hyperlactemia due to toluene sniffing.

## CASE REPORT

**Figure 1:**
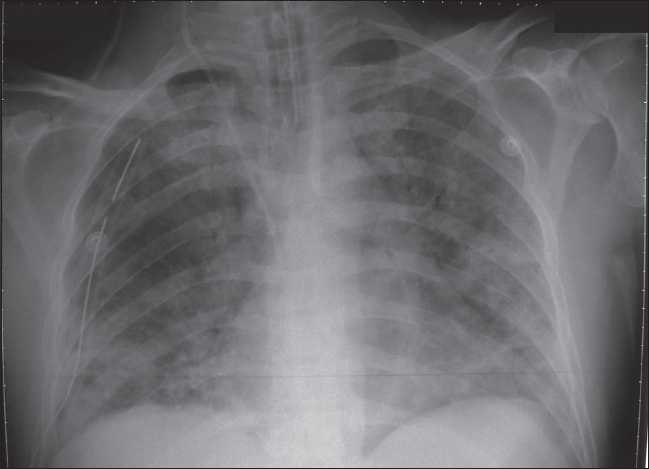
X-ray showed bilateral diffuse infiltrates

A 36-year-old man presented to the emergency department for right-sided abdominal pain. Shortly after arrival, he had a respiratory arrest and required endotracheal intubation and mechanical ventilation. The patient’s family stated that he was a toluene sniffer and he had previous history of admission in an intensive care unit (ICU) with unexplained metabolic acidosis. His comorbid conditions included diabetes, hypertension and renal insufficiency. On physical examination, the patient was hemodynamically unstable with sinus tachycardia (heart rate of 120 beats per minute) and hypotension (blood pressure was 90/45 mmHg) on inotropic medications “norepinephrine started at 10 microgram/min”. He required an FIO_2_of 80% to maintain his oxygen saturation above 90%. He had bilateral coarse crepitations on chest auscultation. Abdominal examinations were unremarkable. Initial arterial blood gases interpretations showed severe mixed acidosis with pH 6.5 (7.35-7.45). His arterial lactate was 16 mmol/L, his CBC was normal, he had a serum creatinine of 211 mmol/L, urea was 50 mmol/L and cardiac enzymes were normal. Toxicology screen was negative for alcohol, salicylates and acetaminophen. Chest radiographs were obtained which showed diffuse bilateral infiltrates [Figure 1]. Compute tomography (CT) of the abdomen and chest showed a small stone in the left kidney and bilateral consolidative changes of both lungs. ECG was normal except for sinus tachycardia. Left ventricular (LV) function was good on echocardiography and chamber sizes were of normal dimensions. Patient received supportive treatment with invasive hemodynamic monitoring, intravenous fluids, vasopressors, mechanical ventilation and empiric antibiotics. Continuous veno-venous hemodialysis was started immediately after admission in ICU. On day 2 his acidosis was improved with arterial pH 7.27, and lactate level dropped to1.5 mmol/L and his FIO_2_ was reduced to 70%. He started to develop rhabdomyolysis with creatine kinase (CK) increase from 470 to 1500 unit/L. Vasopressors were discontinued on the 3^rd^ day and patient was maintained on ventilation and sedation. On the 7th day the patient developed elevated liver enzymes, with alkaline phosphatase 530 unit/L, GGT 402 unit/L, AST (SGOT) 68 unit/L, ALT (SGPT) 47 (unit/L), while his CK normalized. Patient was extubated on the ninth. His urine output and renal functions continuously improved and serum creatinine decreased to 163 mmol/l. He was discharged from the ICU on the tenth day and from the hospital after 15 days in a stable condition.

## DISCUSSION

Hypoxia in patients who abuse inhalants may result from suffocation, particularly from the use of plastic bags, or asphyxia by displacement of oxygen in the alveoli, particularly with butane, isobutane, propane and nitrous oxide.[2] Chemical pneumonitis with surfactant dysfunction bronchospasm and noncardiogenic or hemorrhagic pulmonary edema may occur.[3] This patient developed acute respiratory distress syndrome consistent with the previously published reports.[4–6] Volatile substance use may cause metabolic acidosis, urinary calculi and glomerulonephritis.[3,7] Toluene, in particular, causes metabolic acidosis with profound potassium and phosphate wasting.[8,9] Our patient had history of chronic exposure to toluene and renal insufficiency. A renal stone was evident in the abdominal CT scan. Patients who have an arterial lactate level of more than 5 mmol/L and a pH of less than 7.35 are critically ill and have a very poor prognosis. Multicenter trials have shown a mortality rate of 75% in these patients.[9] Few patients with lactic acidosis survive with a pH less than 6.8.

In our case, initial blood gases showed pH of 6.50 with a lactate level of 16 mmol/L and survived with intensive supportive care.

Toluene use (glue-sniffing) may cause muscle weakness that is associated with elevated CK, hypokalemia, hypophosphatemia and metabolic acidosis with a normal or elevated serum anion gap.[10] It can also result in temporary or progressive cerebellar dysfunction and cranial neuropathies,[1,11] and myocardial depression with decreased heart rate and stroke volume and possible myocardial infarction.[12] Our patient’s CK was abnormal and probably likely due to rhabdomyolysis. His ECG, cardiac enzymes (CK-MB, and troponin I) and echocardiography was not consistent with a cardiac ischemic event.

Chronic exposure to toluene can result in a variety of syndromes, including

1. Generalized muscular weakness (often to the point of quadriparesis) with metabolic acidosis, hypokalemia (with the plasma potassium often being below 2 meq/L), hypophosphatemia, rhabdomyolysis and elevated creatine kinase. These metabolic abnormalities are caused primarily by the conversion of toluene to hippuric acid, with the subsequent rapid excretion of hippurate in the urine.[13]
2. Gastrointestinal complaints, with nausea, vomiting, hematemesis and abdominal pain.[14]
3. Neuropsychiatric symptoms, including peripheral and optic neuropathies, cerebellar ataxia and mental status changes. Permanent cerebellar dysfunction and encephalopathy have been reported.[15]

In conclusion, toluene sniffing is associated with major toxicities including ARDS, acute renal failure and metabolic acidosis with lactate accumulation. Toluene sniffing should be considered in evaluating severe metabolic acidosis. Favorable outcome could be achieved with early diagnosis and proper interventions.

## References

[1] Dinwiddie, SH (1994). Abuse of inhalants: a review. Addiction.

[2] Scalzo AJ, Barkin RM (1997). Inhalation injuries. Pediatric Emergency Medicine: Concepts and Clinical Practice. In.

[3] Espeland K (1995). Identifying the manifestations of inhalant abuse. Nurse Pract.

[4] McManus B, Strange GR, Ahrens W, Lelyveld S, Schafermeyer R (1996). Hydrocarbons. Pediatric Emergency Medicine: A Comprehensive Study Guide.

[5] Carder JR, Fuerst RS (1991). Myocardial infarction after toluene inhalation. Pediatr Emerg Care.

[6] Fitzgerald RL, Fishel CE, Bush LL (1993). Fetality due to recreational use of chlorodifluromethane and chloropentafluromethane. J Forensic Sci.

[7] Kurtzman TL, Otsuka KN, Wahl RA (2001). Inhalant abuse by adolescents. J Adolesc Health.

[8] Streicher HZ, Gabow PA, Moss AH, Kono D, Kaehny WD (1981). Syndromes of toluene sniffing in adults. Ann Intern Med.

[9] Taher SM, Anderson RJ, McCartney R, Popovtzer MM, Schrier RW (1974). Renal tubular acidosis associated with toluene “sniffing". N Engl J Med.

[10] Espeland, K (1995). Identifying the manifestations of inhalant abuse. Nurse Pract.

[11] Neumark YD, Delva J, Anthony JC (1998). The epidemiology of adolescent inhalant drug involvement. Arch Pediatr Adolesc Med.

[12] Carder JR, Fuerst RS (1997). Myocardial infarction after toluene inhalation. Pediatr Emerg Care.

[13] Carlisle EJ, Donnelly SM, Vasuvattakul S, Kamel KS, Tobe S, Halperin ML (1991). Glue-sniffing and distal renal tubular acidosis: Sticking to the facts. J Am Soc Nephrol.

[14] Streicher HZ, Gabow PA, Moss AH, Kono D, Kaehny WD (1981). Syndromes of toluene sniffing in adults. Ann Intern Med.

[15] Brust JC (1993). Other agents. Phencyclidine, marijuana, hallucinogens, inhalants, and anticholinergics. Neurol Clin.

